# Youth engagement in HIV prevention intervention research in sub‐Saharan Africa: a scoping review

**DOI:** 10.1002/jia2.25666

**Published:** 2021-02-10

**Authors:** Sarah E Asuquo, Kadija M Tahlil, Kathryn E Muessig, Donaldson F Conserve, Mesoma A Igbokwe, Kelechi P Chima, Ezienyi C Nwanunu, Lana P Elijah, Suzanne Day, Nora E Rosenberg, Jason J Ong, Susan Nkengasong, Weiming Tang, Chisom Obiezu‐Umeh, Ucheoma Nwaozuru, Yesenia Merino, Titilola Gbaja‐Biamila, David Oladele, Juliet Iwelunmor, Oliver Ezechi, Joseph D Tucker

**Affiliations:** ^1^ Department of Health Behavior Gillings School of Global Public Health University of North Carolina at Chapel Hill Chapel Hill NC USA; ^2^ Department of Epidemiology Gillings School of Global Public Health University of North Carolina at Chapel Hill Chapel Hill NC USA; ^3^ Department of Health Promotion, Education, and Behavior Arnold School of Public Health University of South Carolina Columbia SC USA; ^4^ Clinical Sciences Department Nigerian Institute of Medical Research Lagos Nigeria; ^5^ College of Medicine University of Nigeria Nsukka Nigeria; ^6^ Department of Biochemistry Michael Okpara University of Agriculture Umudike Nigeria; ^7^ College of Medicine Lagos State University Lagos Nigeria; ^8^ Institute for Global Health and Infectious Diseases University of North Carolina at Chapel Hill Chapel Hill NC USA; ^9^ University of North Carolina Project Malawi Lilongwe Malawi; ^10^ Faculty of Infectious and Tropical Diseases London School of Hygiene and Tropical Medicine London UK; ^11^ Central Clinical School Monash University Melbourne Vic. Australia; ^12^ Department of Clinical Research London School of Hygiene and Tropical Medicine London UK; ^13^ Dermatology Hospital Southern Medical University Guangzhou China; ^14^ Department of Behavioral Science and Health Education Saint Louis University Saint Louis MO USA; ^15^ Gillings School of Global Public Health University of North Carolina at Chapel Hill Chapel Hill NC USA; ^16^ Department of Medicine University of North Carolina at Chapel Hill Chapel Hill NC USA

**Keywords:** HIV, youth, engagement, research, Sub‐Saharan Africa

## Abstract

**Introduction:**

Youth engagement in HIV research is generally recognized as essential, but often neglected or minimally implemented in practice. Engagement is a process of working collaboratively with diverse groups of people to address common issues. We conducted a scoping review of youth HIV prevention interventions in sub‐Saharan Africa to identify and categorize forms and levels of youth engagement across the lifespan of intervention research.

**Methods:**

We followed Arksey and O'Malley's framework for organizing a scoping review. We searched seven databases for related articles on identified intervention studies through May 28^th^ 2020. Included studies focused on youth (10 to 24 years old) HIV prevention interventions in sub‐Saharan Africa. Two reviewers independently examined citations and full manuscripts for inclusion. Data were extracted on study characteristics, location, description of youth engagement and extent of engagement. Youth engagement approaches were categorized based on Hart’s ladder as substantial engagement (strong youth decision‐making power), moderate engagement (shared decision making with adults), minimal engagement (no youth decision‐making power) or no engagement.

**Results:**

We identified 3149 citations and included 112 studies reporting on 74 unique HIV interventions. Twenty‐two interventions were in low‐income countries, 49 in middle‐income countries, and three were in both. Overall, only nine interventions (12%) had substantial or moderate youth engagement, two‐thirds (48, 65%) had minimal youth engagement and 17 interventions (23%) had no youth engagement. We also identified specific engagement strategies (e.g. youth‐led research, crowdsourcing) that were feasible in multiple settings and resulted in substantial engagement.

**Conclusions:**

We found limited youth engagement in youth HIV prevention intervention studies in sub‐Saharan Africa. However, several activities resulted in substantial youth engagement and could be relevant in many low‐and‐middle‐income‐country (LMIC) settings.

## 1 INTRODUCTION

Young people (15‐24 years old) in sub‐Saharan Africa accounted for 19% of the estimated 1.7 million new HIV infections globally in 2019, whereas adolescents (10 to 19 years old) in the region made up 8% of total new HIV infections [1, 2]. The number of young people in Africa is estimated to increase by 42% by 2030 [3]. The demographic youth bulge in Africa suggests that HIV prevention will continue to be a critical issue in the coming years. However, similar to other low and middle income countries (LMICs), African nations may have fewer formal and informal mechanisms for stakeholder engagement [4]. We define engagement as a process of working collaboratively with diverse groups of people to address common issues [5, 6].

Youth engagement is essential for effective intervention development. The Joint United Nations Programme on HIV/AIDS (UNAIDS), the United States Agency for International Development (USAID), and other organizations encourage youth engagement in the development of HIV interventions [7, 8]. Youth engagement in HIV interventions increases HIV knowledge, reduces HIV stigma, and facilitates behaviour change [8, 9]. In the context of research, youth engagement can enhance recruitment, create more youth‐friendly interventions and promote dissemination and sustainability [10, 11]. While many studies have examined the effectiveness of youth HIV interventions in sub‐Saharan Africa [8, 12, 13], the extent to which youth are engaged at various stages of intervention research activities (pre‐intervention, intervention and post‐intervention) is rarely explored. Furthermore, the measurement of youth engagement is not standardized. Categorizing levels of youth engagement across study phases will help identify important gaps in the research process, while describing types of engagement approaches that have been used in partnership with youth, providing a resource for HIV prevention research.

The purpose of this study was to categorize and determine the extent of youth engagement in HIV prevention research in sub‐Saharan Africa using a scoping review. We chose a scoping review because of the following reasons: youth engagement strategies were not sufficiently similar to allow pooling; we are not examining the effect of an intervention on an outcome and this prevented assessment of risk of bias; substantial heterogeneity in key operational definitions; identifying research gaps in the existing literature may be well addressed through a scoping review [14].

## 2 METHODS

### 2.1 Search strategy

We conducted a scoping review of published literature based on Arksey and O’Malley’s framework for conducting scoping reviews [14]. Scoping reviews examine the extent, range, and nature of research activity for a given topic [14]. On January 15, 2020, we searched five medical research databases (PubMed, Global Health, Scopus, Embase and Cochrane), one clinical trial database (ClinicalTrials.gov), and one non‐peer‐reviewed literature source (Open Gray). Included publications were HIV prevention studies in sub‐Saharan Africa focused on youth (aged 10 to 24 years). The search strategy included variations of the following terms: stakeholder engagement, youth, HIV and low‐and‐middle‐income countries (defined per World Bank guidance) [15]. We exported the records from our search and removed duplicates using Covidence, an online article screening, and data extraction programme.

### 2.2 Study selection

Inclusion criteria were behavioural and biomedical research studies with human subjects conducted between January 2000 and January 2020, focused primarily (>50%) on youth, related to HIV prevention intervention, and conducted in sub‐Saharan Africa. Studies with stakeholder engagement in the manuscript but without youth engagement were still included. We excluded records that were focused on secondary prevention for youth living with HIV; were cross‐sectional or observational; were systematic or narrative reviews; were secondary data analyses; or were not written in English.

SA and KMT independently reviewed titles and abstracts for inclusion, and KM and DC resolved any discrepancies when needed. Following the title and abstract screening phase, SA and KMT conducted independent full‐text reviews, further excluding studies based on our pre‐established criteria. Data extracted included a description and degree of youth engagement in each study (described below), country, intervention type (behavioural, biomedical or both) and gender of study participants.

The unit of analysis in this review was youth HIV prevention intervention studies. However, descriptions of these interventions were published in more than one journal article. Thus, after identifying the final set of interventions to include using the search strategy described above, we conducted a secondary search for all related articles describing these interventions to ensure that we holistically captured available data on youth engagement. We searched PubMed and ClinicalTrials.gov on 15 January 2020 using search terms gleaned from article abstracts describing our final set of included interventions (e.g. study acronyms, names of study groups, clinical trial registration numbers). From the resulting related articles, we repeated the process of data extraction pertaining to youth engagement activities in our included intervention studies. We updated our search on May 28 2020.

### 2.3 Categorizing engagement

**Figure 1:**
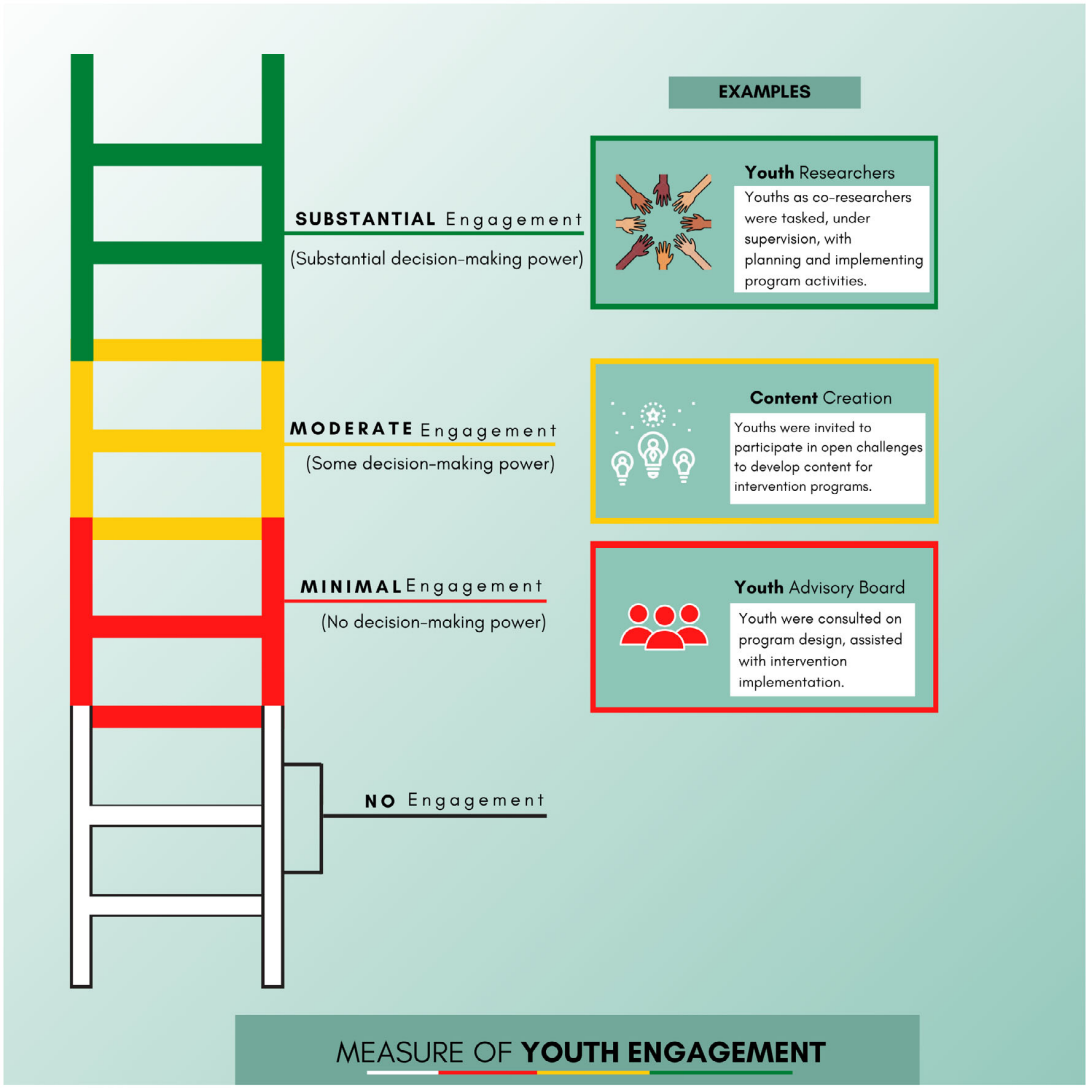
Categorizing youth engagement, adapted from Hart’s ladder [16].

We used Hart’s ladder to specify the extent of youth engagement [16]. Hart’s ladder is a typology that describes different degrees of youth engagement in projects or programmes. It has eight steps, which progress in a bottom to top fashion, from no engagement to different degrees of engagement. We modified Hart’s ladder by grouping the steps with youth engagement into substantial, moderate, minimal and no youth engagement, based on the decision‐making power of the youth in the research study (Figure 1). Substantial youth engagement was defined as research activities that were youth‐initiated and directed. Adults either created an enabling environment or made relevant contributions, with youth having substantial decision‐making power and opportunities for youth leadership. Moderate youth engagement was defined as adult‐initiated activities with shared decision making between youth and adults. Minimal youth engagement was defined as youth being consulted to get their opinions, assigned specific roles or informed about events surrounding research activities, without any decision‐making power. No youth engagement was defined as the absence of participatory approaches or activities during research. We assumed that meaningful youth engagement would be described in the research study.

### 2.4 Data analysis

We used thematic analyses to summarize textual data describing various youth engagement activities employed in identified research studies and were then classified using a conceptual framework based on our modified Hart’s ladder. Two researchers (SA and KMT) independently analysed textual data into the four categories. Engagement activities identified were then categorized and independently coded once for each intervention as per the modified Hart’s ladder described above. We used the University of Witwatersrand Reproductive Health and HIV Institute (Wits RHI) Good Participatory Practice Implementation Model, adapted from the UNAIDS Good Participatory Practice Guidelines to categorize the timing of engagement activities as pre‐intervention, intervention or post‐intervention research phases [7, 17]. Pre‐intervention phase referred to planning and readiness activities, including stakeholder advisory mechanisms, protocol development, ethical approval, field testing and related formative research activities. Intervention phase referred to activities during the actual implementation of the HIV prevention intervention studies. Post‐intervention phase referred to dissemination, results reporting and related activities.

SA and KMT conducted data extraction for all identified studies for the review. To ensure consistency in coding, they first independently extracted and coded ten studies and then assessed their process for intercoder reliability by dialogue. This ensured standardization of extracted data for categorizing the studies and minimized the risk of misclassification. After the two reviewers concluded the process for checking for intercoder reliability the remaining selected studies were then divided evenly between SA and KMT for independent data extraction. Each study was given a score for degree of engagement at each research phase based on the coded engagement activities. An overall assessment was made for each study based on the research phase with the highest score for that study. Given the substantial heterogeneity in key operational definitions of engagement [18], we did not pool findings. We did not require an ethical board review for this scoping review study.

## 3 RESULTS

**Figure 2:**
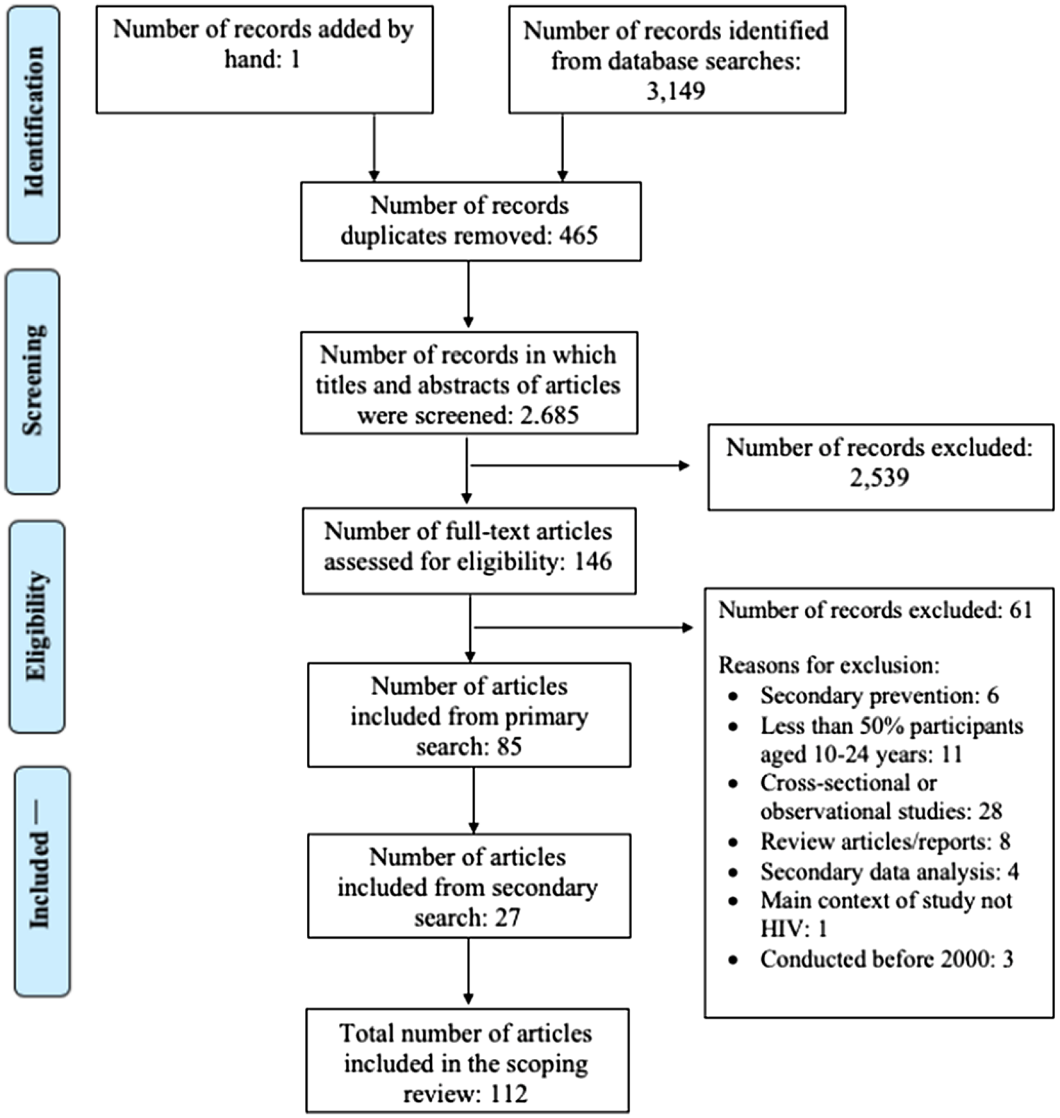
Flow chart of included studies in this scoping review.

Our search strategy yielded 3149 citations and 2684 unique citations. Aside from the citations identified through our database searches, one study was added by hand. After excluding ineligible citations, we examined 146 full‐text manuscripts. Of these, 85 manuscripts met the eligibility criteria and were included (Figure 2). Following our secondary search for studies that described the interventions identified, we found 27 additional manuscripts. Thus, the total number of manuscripts reviewed was 112 [19, 20, 21, 22, 23, 24, 25, 26, 27, 28, 29, 30, 31, 32, 33, 34, 35, 36, 37, 38, 39, 40, 41, 42, 43, 44, 45, 46, 47, 48, 49, 50, 51, 52, 53, 54, 55, 56, 57, 58, 59, 60, 61, 62, 63, 64, 65, 66, 67, 68, 69, 70, 71, 72, 73, 74, 75, 76, 77, 78, 79, 80, 81, 82, 83, 84, 85, 86, 87, 88, 89, 90, 91, 92, 93, 94, 95, 96, 97, 98, 99, 100, 101, 102, 103, 104, 105, 106, 107, 108, 109, 110, 111, 112, 113, 114, 115, 116, 117, 118, 119, 120, 121, 122, 123, 124, 125, 126, 127, 128, 129, 130]. These manuscripts described 74 unique intervention research studies (Table 1) [19, 20, 21, 22, 23, 24, 25, 26, 27, 28, 29, 30, 31, 32, 33, 34, 35, 36, 37, 38, 39, 40, 41, 42, 43, 44, 45, 46, 47, 48, 49, 50, 51, 52, 53, 54, 55, 56, 57, 58, 59, 60, 61, 62, 63, 64, 65, 66, 67, 68, 69, 70, 71, 72, 73, 74, 75, 76, 77, 78, 79, 80, 81, 82, 83, 84, 85, 86, 87, 88, 89, 90, 129, 130]. Of the 74 unique intervention studies identified, 72 were solely behavioural interventions and two had both behavioural and biomedical components that included HIV, sexually transmitted infections (STI) and pregnancy screening. Thirty‐five interventions were conducted in southern Africa, 22 in East Africa, 12 in West Africa, two in Central Africa and three were multi‐regional. Twenty‐two intervention studies were in low‐income countries, 49 in middle‐income countries and three in both country income levels. Sixty‐three interventions focused on populations with male and female genders, nine focused only on women and two focused solely on men. Thirty‐seven interventions were conducted before 2010 (Table S1) and 37 interventions were conducted after 2010 (Table S2). Most interventions with youth engagement used multiple engagement approaches at different phases of research (Table 2).

**Table 1:** Characteristics of youth HIV prevention interventions in sub‐Saharan Africa between 2000 to 2020 (N = 74)

	n	%
Region		
Central	2	2.7
East	22	29.7
South	34	45.9
West	13	17.6
Multi‐region	3	4.1
Country income level^a^		
Low income	22	29.7
Middle income	49	66.2
Both	3	4.1
Gender of participants		
Only women	9	12.2
Only men	2	2.7
All genders	63	85.1
Intervention type^b^		
Behavioural	72	97.3
Behavioural and biomedical	2	2.7

^a^ Based on World Bank criteria

^b^ there were no solely biomedical interventions.

**Table 2:** Examples of youth engagement approaches used at each phase of youth HIV prevention intervention research in sub‐Saharan Africa from 2000 to 2020 (N = 74)

	Pre‐intervention phase	Intervention phase	Post‐intervention phase
Substantial youth engagement: youth‐initiated activities	Trained youth researchers initiated and planned intervention research	Trained youth researchers implemented intervention research	Trained youth researchers analysed research findings
	Crowdsourcing^a^ open call		Youth initiated post‐intervention sustainability activities
Moderate youth engagement: adult‐initiated shared decision making with youth	Translated intervention components to youth‐acceptable language	Media and content creation Facilitated research activities Developed and delivered drama performances Developed workplans for lectures and community outreaches Led health seminars and key intervention components	Intervention data dissemination
Minimal youth engagement: no decision‐making power	Youth/Community advisory boards Focus group discussions Qualitative interviews Surveys Photovoice	Consulted for programme adaptation during on‐going implementation	Focus group discussions Qualitative interviews Surveys

^a^ Crowdsourcing is the process of having a group solve a problem whose solution has public benefit; this solution is then shared widely with the public [131].

### 3.1 Overall assessment

**Figure 3:**
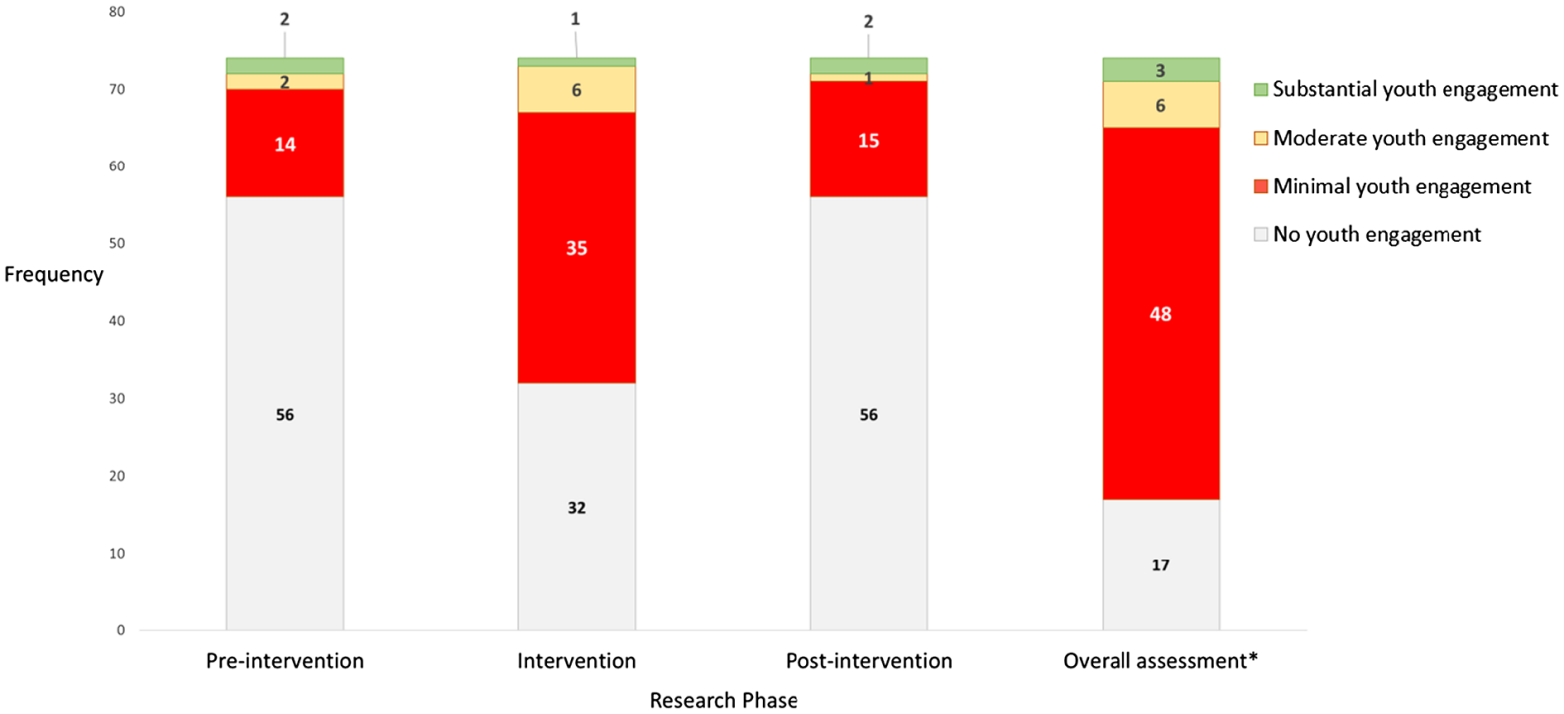
Degree of youth engagement in HIV prevention interventions at different phases of research in sub‐Saharan Africa from 2000 to 2020 (N = 74). Substantial youth engagement: youth‐initiated activities; Moderate youth engagement: shared decision making with adults; Minimal youth engagement: no decision‐making power. *An overall assessment was made for each intervention based on the research phase with the highest level of engagement.

Three intervention studies (4%) had substantial engagement in at least one research phase whereby youth initiated and carried out some research activities from start to finish (Figure 3, Video S1 abstract) [19, 20, 21]. Of these three interventions, one had substantial youth engagement in all three phases of research [20]. For this intervention, street‐connected youth peer educators were trained to conduct research. These peer educators or youth researchers initiated, planned, and implemented a series of HIV prevention programme activities within their communities that targeted similar groups of youth, and carried out data analysis of their programme effectiveness [20]. Substantial youth engagement was also identified with engagement approaches that used crowdsourcing of ideas for interventions [21], and youth‐initiated post‐intervention community HIV prevention effort [19]. Crowdsourcing is the process of having a group solve a problem whose solution has public benefit; this solution is then shared widely with the public [131].

Moderate youth engagement was identified in six interventions (8%) whereby youth, under supervision, were empowered to decide how to deliver intervention components, implying shared decision making with adults (Video S1 abstract) [22, 23, 24, 25, 26, 27]. Most interventions (48, 65%) had minimal engagement, with youth having no decision‐making power [28, 29, 30, 31, 32, 33, 34, 35, 36, 37, 38, 39, 40, 41, 42, 43, 44, 45, 46, 47, 48, 49, 50, 51, 52, 53, 54, 55, 56, 57, 58, 59, 60, 61, 62, 63, 64, 65, 67, 68, 69, 70, 71, 72, 75, 87, 90, 130], and some interventions (17, 23%) had no engagement at any phase of research [66, 88, 89, 129].

We identified two studies which utilized digital and social media‐based interventions or mHealth [21, 90]. Of these two studies, one was categorized as having substantial youth engagement, whereas the other had minimal youth engagement. Both studies were conducted after 2010. We also assessed of the extent of youth engagement over time by comparing engagement in studies conducted in or before 2010 [19, 20, 22, 34, 39, 40, 41, 45, 46, 47, 48, 52, 53, 54, 59, 60, 66, 68, 72, 73, 77, 78, 80, 82, 83, 88, 89, 109, 115, 121, 130], to studies conducted after 2010 [21, 24, 28, 29, 30, 42, 43, 44, 50, 51, 56, 57, 58, 60, 62, 63, 64, 67, 69, 70, 71, 75, 76, 79, 81, 84, 85, 86, 90, 100, 110, 111, 129]. We found youth engagement in 28 out of 37 interventions (76%) conducted in or before 2010, and in 30 out of 37 interventions (81%) conducted after 2010.

### 3.2 Pre‐intervention phase

We identified two interventions with substantial youth engagement at the pre‐intervention phase of research (Figure 3) [20, 21]. The first was a crowdsourcing open call for ideas on HIV self‐testing delivery methods focused on engaging youth [21]. In the second study, street‐connected youth peer educators initiated and planned a programme of activities within their Non‐Governmental Organization (NGO) including regular HIV prevention clubs, individual counselling and seminars, with support from NGO staff. They also developed post‐intervention survey questionnaires for their research [20].

Two interventions had moderate youth engagement at the pre‐intervention research phase [23, 24]. In the first intervention, youth researchers carried out unstructured observations, facilitated informal discussion groups with community members, and worked with community members to highlight components of the intervention research that were important to their community. In this way, youth helped direct the research objectives [23]. In the second study, street‐connected youth peer educators adapted and translated intervention components into terms that similar youth use and comprehend [24]. The youth also nominated representatives to engage in focus group discussions, and elected representatives who communicated ideas and concerns to the study team regarding the proposed programmes [24].

Fourteen interventions (19%) had minimal youth engagement at the pre‐intervention phase [28, 31, 33, 35, 36, 43, 45, 49, 54, 59, 60, 67, 71, 87]. These interventions used youth advisory boards or committees (3, 4%) [59, 67], photovoice (1, 1%) [28], focus group discussions (10, 14%) [28, 31, 33, 35, 43, 45, 54, 60, 71, 87], qualitative interviews (4, 5%) [36, 43, 60, 87], and surveys (3, 4%) [28, 45, 49], to get youth’s views and opinions, or areas of focus for intervention. In as much as the youth were consulted through these aforementioned mechanisms, it was unclear the extent to which youth opinions shaped final decisions with regards to research components or outcomes.

About three quarters of studies (56, 76%) had no youth engagement at the pre‐intervention phase [19, 22, 29, 30, 32, 44, 46, 47, 48, 50, 51, 52, 53, 55, 56, 57, 58, 61, 62, 63, 64, 65, 66, 68, 69, 70, 73, 74, 75, 76, 77, 78, 79, 80, 81, 82, 83, 84, 85, 86, 88, 89, 90, 129, 130].

### 3.3 Intervention phase

The intervention phase had the highest number of interventions with youth engagement (42, 57%) [19, 20, 22, 23, 24, 25, 26, 27, 28, 29, 30, 31, 32, 33, 34, 39, 42, 43, 44, 45, 46, 47, 49, 51, 52, 53, 56, 57, 58, 59, 61, 63, 64, 65, 67, 68, 69, 70, 71, 72, 75]. There was one intervention study with substantial youth engagement at this phase of research. In the study with street connected youth, substantial engagement continued from the pre‐intervention to the intervention phase. These youth implemented their planned programme of activities that targeted other street‐connected youth in their communities [20].

There were six studies (8%) with moderate youth engagement at the intervention phase [19, 22, 23, 25, 26, 27]. All six studies utilized trained peer educators who were empowered to decide how to deliver intervention components, implying shared decision making with adults on intervention implementation. In one intervention, peer educators developed their workplans for class‐based lectures and community outreaches [22]. In two other interventions, both school‐based, peer educators led key intervention components including seminars and health education activities, informal group discussions, individual counselling, drama, songs and other performances [26, 27]. Another study, which was also school‐based, had peer educators lead student clubs in which members of the clubs created songs, videos, journalistic articles and other types of media that promoted knowledge and dialogue related to HIV, as well as encouraged peers to get tested. The best media content created were selected to be used for a city‐wide post‐intervention HIV campaign [25]. One intervention had drama performances by the peer educators as its main component, with youth playing a major role in directing drama content [19]. In another intervention (described earlier as having moderate youth engagement at pre‐intervention phase), youth researchers guided and facilitated research activities initially agreed upon with community members [23].

All interventions assessed as having minimal youth engagement at the intervention phase (35, 47%) utilized trained peer educators [24, 28, 29, 30, 31, 32, 33, 34, 42, 43, 44, 45, 46, 47, 49, 51, 52, 53, 56, 57, 58, 59, 61, 63, 64, 65, 67, 68, 69, 70, 71, 72, 75]. In all of these interventions, peer educators were assigned to lead discussions or teach their peers about high risk and protective behaviours related to HIV and sexual/reproductive health; the peer educators did not modify the interventions in any way or have any decision‐making power during intervention implementation. Two of these interventions also involved peer educators in drama performances or role plays, although it was not reported that peer educators had any say in the performance content [45, 53].

There were 32 interventions (43%) with no youth engagement approach used at the intervention phase [21, 35, 36, 37, 38, 54, 55, 60, 66, 73, 74, 76, 77, 78, 79, 80, 81, 82, 83, 84, 85, 86, 87, 88, 89, 90, 129, 130].

### 3.4 Post‐intervention phase

There were two interventions with substantial youth engagement at the post‐intervention phase [19, 20]. In one study – described earlier as having substantial engagement at pre‐intervention and intervention research phases – street‐connected youth peer educators carried out data analysis to assess the effectiveness of their programme and determined areas for improvement [20]. In the second intervention with substantial engagement at the post‐intervention phase, peer educators initiated and organized post‐intervention community HIV testing and counselling health fairs in conjunction with district health officials, following survey responses from community members who attended their drama performances [19]. This study also had moderate youth engagement at the intervention phase.

One intervention had moderate youth engagement post‐intervention, with shared decision making with adults [23]. In this intervention – also described earlier as having moderate engagement at both pre‐intervention and intervention phases – youth researchers discussed research findings with their community during a one‐day report‐back session [23].

Interventions with minimal youth engagement at the post‐intervention phase (15, 20%) [22, 24, 28, 34, 37, 38, 40, 41, 43, 44, 48, 50, 55, 90, 130] used different engagement approaches to get youth’s opinions and feedback about the intervention and study components such as surveys (4, 5%) [24, 34, 44, 55], focus group discussions (13, 18%) [22, 24, 28, 37, 38, 40, 41, 44, 48, 50, 90, 102, 132], and individual qualitative interviews (3, 4%) [28, 44, 102]. Two studies assigned peer educators to collect data by administering structured questionnaires to study participants post‐intervention [24, 55]. In these interventions with minimal youth engagement, youth had no described decision‐making power to determine post‐intervention phase outcomes.

About three‐quarters of studies (56, 76%) had no youth engagement activity at the post‐intervention phase [21, 25, 26, 27, 29, 30, 31, 32, 33, 35, 36, 39, 42, 45, 46, 47, 49, 51, 52, 53, 54, 56, 57, 58, 59, 60, 61, 62, 63, 64, 65, 66, 67, 68, 69, 70, 71, 72, 73, 74, 75, 76, 77, 78, 79, 80, 81, 82, 83, 84, 85, 86, 87, 88, 89, 129].

## 4 DISCUSSION

This scoping review describes the extent of youth engagement in HIV prevention interventions in sub‐Saharan Africa. Most interventions had minimal or no youth engagement. Despite the recognized importance of youth engagement in HIV research [133, 134], prior youth HIV literature reviews have not focused on engagement of youth, but rather on youth as recipients of interventions [12, 135], or on evaluating peer‐led programmes among youth [135, 136]. This scoping review extends the literature by measuring the extent of youth engagement, focusing on engaging sub‐Saharan African youth, and rigorously examining youth engagement in selected interventions.

We found that youth engagement was minimal or absent in many youth HIV prevention studies from sub‐Saharan Africa. This finding is consistent with a broader literature suggesting that youth are often excluded from meaningful engagement in HIV interventions [137, 138]. This finding suggests that while youth frequently participate in the research process, they are not often engaged in activities that share decision‐making power with adults or provide opportunities for youth leadership. This may be related to ethical concerns about the competing demands among youth [139], lack of youth training and capacity building opportunities [137, 140], or adult perceptions about limited youth capacity [141]. It may also be associated with a lack of parental consent; given the stigmatized nature of HIV, parents may have more concerns allowing their youth to engage in HIV prevention research than other less stigmatized health interventions [142]. Additionally, studies may not have the funding or time necessary for robust youth engagement . However, there were some examples of substantial engagement in which youth extensively developed, implemented and analysed interventions. This suggests that there are feasible opportunities for considerable youth engagement at all phases of intervention research.

We identified two creative ways to engage youth in HIV prevention research. One intervention held a crowdsourcing open call for youth to share their ideas on how to promote HIV self‐testing among youth [21]. This intervention engaged a large number of youth with a diverse set of ideas on HIV interventions that are relevant to their needs. This crowdsourcing approach has been used in other LMIC settings [143]. Other interventions identified in this review described youth as co‐researchers who were tasked, under supervision, with planning and implementing programme activities, disseminating research findings or organizing post‐intervention community HIV testing and counselling [19, 20, 23]. Crowdsourcing and youth as co‐researchers are mechanisms that foster youth engagement in the HIV research process. These participatory approaches provide an environment for meaningful youth engagement, which can lead to the development of health services that are appropriately tailored to the needs of youth.

The review had several limitations. First, our search strategy included “engagement” in the terms. As a result, we likely over‐estimated the extent of youth engagement in HIV prevention research in sub‐Saharan Africa. Second, some studies may not have described youth engagement. However, we also did a secondary search to identify additional manuscripts related to the same intervention. In addition, research checklists [144], best practice statements [145, 146], and guidelines [7], underline the importance of reporting youth engagement in HIV research. Third, our review does not capture HIV interventions with youth engagement described in non‐English journals. While a limitation, evidence suggests that excluding non‐English studies does not impact review findings from systematic reviews [147, 148]. Fourth, youth engagement categorization was only done by one reviewer for each intervention. There is a risk for misclassification bias. In order to reduce this bias, the two coders created standardized criteria and compared notes on the same ten studies. Fifth, we did not address efficacy of the analysed studies in our review. This is because the purpose of our review was to not to assess the efficacy of engagement, but to simply describe the extent of youth engagement in HIV studies.

Findings from this review have public health and programmatic implications. There is a disconnect between advocacy for meaningful youth engagement and current youth engagement in practice. HIV interventions can fill this gap by using creative approaches to meaningfully engage youth at all phases of the research process. These creative approaches can include opportunities for youth to create and lead HIV interventions in their communities. From a policy perspective, youth engagement may improve the process of developing new programmes for youth [149]. Policymakers should be informed by the needs of youth, which can best happen when youth are engaged in the HIV research process [149]. Moreover, youth may need more training and support in order to have greater power in decision making related to research studies. It is important that efforts to increase capacity‐building and provide mentorship to youth are considered during the design of studies, when research priorities are being established. Guidance on ethical issues [150] related to appropriate youth engagement may also facilitate this process. Finally, research that demonstrates the value of additional engagement is needed. There is a compelling scientific rationale for youth engagement that could serve as the foundation for further studies.

## 5 CONCLUSIONS

Our scoping review shines a light on how youth engagement can transform both the process and outputs of HIV research. In terms of process, our co‐authorship team included four youth researchers who each made unique and valuable contributions, underlining the benefit of robust youth inclusion. Our youth co‐authors led the development of the infographic and video, steering the scoping review towards a younger readership. Researchers describing HIV research studies should explicitly consider youth research audiences in order to make research findings relevant to youth. At the same time, strong youth engagement will require researchers to not only trust youth and give them agency, but to provide the mentorship and support necessary to achieve these goals. The studies including digital youth engagement also demonstrate how youth engagement can benefit the outputs of HIV research, developing innovative interventions, services, and approaches. One of the silver linings of COVID‐19 may be to accelerate digital engagement in HIV studies. The expanding opportunity for digital youth engagement merits further research and action.

## COMPETING INTEREST

The authors declare that they have no conflict of interest.

## AUTHORS’ CONTRIBUTIONS

All authors substantially contributed to project conception, reviewed edited the manuscript, gave final approval of the version to be published and agreed to be accountable for all aspects of this review. SA and KMT: led the scoping review process (project conceptualization, developing and implementing the search strategy, title, abstract and full‐text review, data extraction, writing the manuscript). KM: project conceptualization, developing the search strategy, full‐text review, reviewing and editing the manuscript. DC: project conceptualization, developing the search strategy, title and abstract review, reviewing and editing the manuscript. MAI and KPC: developing the video abstract, reviewing and editing the manuscript. ECN and LPE: developing the infographic (Figure 1), reviewing and editing the manuscript. SD: conducting secondary search of studies, reviewing and editing the manuscript. NER, JJO, SN, WT: project conceptualization, developing the search strategy, reviewing and editing the manuscript. CO, UN, YM, TG, DO, JI, OE: data interpretation, reviewing and editing the manuscript. JDT: project conceptualization, overall guidance and oversight of the scoping review process, reviewing and editing the manuscript.

## Supporting information


**Table S1.** HIV prevention interventions in sub‐Saharan Africa conducted before 2010.

**Table S2.** HIV prevention interventions in sub‐Saharan Africa conducted after 2010.

**Video S1.** Video abstract of the scoping review. Link available here: https://drive.google.com/file/d/1Dxxzt89PmHTkV7E‐ihjeZDeh5‐I53tba/view
